# An Integrated Approach Based on Multiplexed Protein Array and iTRAQ Labeling for In-Depth Identification of Pathways Associated to IVF Outcome

**DOI:** 10.1371/journal.pone.0077303

**Published:** 2013-10-16

**Authors:** Valeria Severino, Livia Malorni, Anna Emilia Cicatiello, Vittoria D’Esposito, Salvatore Longobardi, Nicola Colacurci, Nadia Miraglia, Nicola Sannolo, Annarita Farina, Angela Chambery

**Affiliations:** 1 Department of Environmental, Biological and Pharmaceutical Sciences and Technologies, Second University of Naples, Caserta, Italia; 2 Institute of Biostructures and Bioimaging-IBB, CNR, Napoli, Italia; 3 Department of Experimental Medicine, Section of Hygiene, Occupational Medicine and Forensic Medicine, School of Medicine, Second University of Naples, Napoli, Italia; 4 Department of Translational Medical Sciences, Federico II University of Naples, Napoli, Italia; 5 Medical Department, MerckSerono S.p.A, Roma, Italia; 6 Department of Obstetrics, Gynaecology and Reproductive Sciences, Second University of Naples, Napoli, Italia; 7 Biomedical Proteomics Research Group, Department of Bioinformatics and Structural Biology, Geneva University, Geneva, Switzerland; 8 CIRPEB-Centro Interuniversitario di Ricerca sui Peptidi Bioattivi, Napoli, Italia; University of South Florida College of Medicine, United States of America

## Abstract

The emergence of high-throughput protein quantification methodologies has enabled the comprehensive characterization by longitudinal and cross-sectional studies of biological fluids under physiological and pathological conditions. In particular, the simultaneous investigation of cytokines and growth factors signaling pathways and their associated downstream effectors by integrated multiplexed approaches offers a powerful strategy to gain insights into biological networks and processes in living systems. A growing body of research indicates that bioactive molecules of human reproductive fluids, including human follicular fluid (hFF), may affect oocyte quality, fertilization and embryo development, thus potentially influencing the physiopathology of pregnancy-related conditions.

In this work, an iTRAQ labeling strategy has been complemented with a multiplexed protein array approach to analyze hFFs with the aim to investigate biological processes and pathways related to in vitro fertilization (IVF) outcome. The iTRAQ labeling strategy lead to the quantification of 89 proteins, 30 of which were differentially expressed in hFFs with successful compared to unsuccessful IVF outcome. The targeted study, based on multiplexed antibody protein arrays, allowed the simultaneous quantification of 27 low abundance proteins, including growth factors, chemokines and cytokines endowed with pro- and anti-inflammatory activity. A significant number of differentially regulated proteins were involved in biological functions related to blood coagulation, acute phase response signaling and complement system. Overall, the present results provide an integrated overview of protein changes in hFFs associated to IVF outcome, thus improving current knowledge in reproductive medicine and fertility research.

## Introduction

The application of proteomic technologies in reproductive medicine and pregnancy research is rapidly expanding [1-4]. Human follicular fluid (hFF) is one of the most important body fluids related to reproduction since it represents the *in vivo* microenvironment for the oocyte maturation [5]. During folliculogenesis, regulatory factors are secreted by granulosa cells or are derived from the theca capillaries. The blood–follicular barrier also influences the composition of hFF by the selective transfer of specific factors including proteins, anticoagulants and enzymes. The fine balance of these substances is recognized to play a significant role in supporting female fertility. Therefore, hFF represents a unique reservoir of potential biomarkers for the assessment of the oocyte and embryo quality of relevance for pregnancy outcome of *in vitro* fertilization (IVF) treatment [3].

For these reasons, the proteomic profiling of hFF has become an active area of research, especially in the last decade. Proteomic strategies for the analysis of reproductive fluids can be grouped in two main categories, namely qualitative surveys for proteome profiling and comparative approaches for differential protein detection. 

In a pioneering study by Anahory and coworkers, the proteomic profile of hFFs from women undergoing IVF was evaluated by two dimensional gel electrophoresis (2-DE) and matrix-assisted laser desorption/ionization time-of-flight (MALDI-TOF) mass spectrometry (MS), leading to the identification of three proteins (i.e. thioredoxin peroxidase 1, transthyretin, and retinol-binding protein) as novel components of hFF [6]. Since then, similar proteomic methods have been exploited to investigate the protein or peptide profiles of hFFs as potential predictors of oocyte growth and maturation [7-11]. Recently, a methodological improvement has been reported for the characterization of hFFs by 2-DE [12]. Furthermore, a shotgun MS approach based on the direct analysis of proteolytic peptides by liquid chromatography tandem MS (LC-MS/MS) has been applied to the screening of hFFs [13].

Although the characterization of proteins present in hFF is essential for determining the molecular environment surrounding oocyte during its maturation, a step toward comprehension of its influence on oocyte quality and IVF outcome should necessarily involve the setting up of quantitative comparative approaches to obtain differential proteomic profiles. Despite the wide application of proteomic strategies in reproductive research, to date, few comparative studies have been performed on hFF. Kim et al. identified fibrinogen γ and anti-thrombin as differentially expressed proteins in hFFs of women with recurrent spontaneous abortion, thus hypothesizing that coagulation factors may play an important role in maintaining normal pregnancy [14]. A paired comparison of protein profiles of hFFs with plasma/serum of women undergoing successful IVF also revealed differences in proteins belonging to the innate immune system and complement cascade of potential importance in reproductive processes [15]. Furthermore, an investigation for defining the protein and steroid profiles in hFFs potentially associated with IVF outcome has been reported by Kushnir and coworkers [16]. In this study, hFFs characterization was accomplished by depletion of abundant proteins, pre-fractionation by ultrafiltration and LC-MS/MS analysis followed by a semi-quantitative analysis using spectral counting. 

Although the proteomic surveys on hFF are beginning to deepen the current knowledge of candidate factors relevant for oocyte quality evaluation, the delineation of networks and pathways involved into the physiology of reproduction and pathological conditions of infertility is still challenging. The huge dynamic range of protein concentrations in biological matrices prevents the establishment of connections between medium/high and low abundant proteins. This aspect is even more important since low abundance proteins (e.g. cytokines, chemokines and growth factors) are key modulators of cellular pathways acting as upstream signals triggering specific responses. Given the current technical limitations of proteomic technologies, experimental strategies based on multiplexed protein measurements by using complementary methodologies are rapidly increasing for descriptive as well as predictive biological research.

In this work, an integrated approach based on multiplexed flow cytometric protein array and quantitative iTRAQ proteomic has been exploited for the comparative evaluation of hFFs based on IVF outcome.

## Materials and Methods

### Ovarian stimulation protocols and patients enrollment

hFFs were sampled from 12 women undergoing IVF treatment for tubal or male infertility at the Center of Reproductive Medicine, Department of Obstetrics and Gynecology of the Second University of Naples, Italy. Patients with polycystic ovaries (PCO), endometriosis or unexplained infertility were excluded from the study. All female patients gave their informed consent prior to sample collection. Ovarian stimulation was achieved by the administration of recombinant follitropin alfa and GnRH analogues (agonists and antagonists). The starting dose was from 150 U.I. to 225 U.I. depending on the woman’s age and/or ovarian reserve estimated. The further dose was personalized according to the individual response. The ovarian response was monitored by serum estradiol assays and vaginal ultrasonographic scans of follicles.

Oocyte maturation was triggered by hCG 10.000 U.I. and the pick up was performed 34-36 h after hCG administration, by vaginal route. After oocyte isolation, only macroscopically clear fluids, indicating lack of blood contamination, were considered in the study. hFFs were centrifuged at 3000 g at 4°C for 15 min to remove cellular components and debris and then transferred into sterile polypropylene tubes and frozen at -80 °C. hFFs were grouped in two categories on the basis of the positive or negative IVF outcome (FF+ and FF-, respectively). In particular, for each group were selected six hFF samples from women who achieved pregnancy and six samples, as control group, from women who didn’t achieve pregnancy.

### Sample preparation

Aliquots of hFF samples (10 µL) were depleted of abundant proteins (i.e. Human serum albumin and the major subclasses of IgG) using the ProteoSeek Albumin/IgG Removal kit (Pierce, Rockford, IL, USA) according to manufacturer’s recommendations. Equal amounts of proteins (50 μg) from each sample were pooled based on IVF outcome (pFF+, pregnancy; pFF-, no pregnancy) and desalted by using Micro DispoDialyzer devices with a 5 kDa cut-off (Harvard apparatus, Holliston, MA, USA) according to manufacturer’s instructions. Protein concentration of pooled samples was determined by the Bradford method, according to manufacturer’s instructions (Biorad, Milan, Italy).

### In-solution tryptic digestion

Equal aliquots of proteins (60 μg) from pFF+ and pFF- samples were lyophilized and resuspended in 100 µL of 0.1 M triethylammonium hydrogen carbonate (TEAB) buffer pH 8.0. An equal amount (1 μg) of bovine β-Lactoglobulin (LACβ) was spiked in each sample to serve as an internal standard for experimental bias correction. Proteins were reduced by adding 1 µL of 1% SDS and 2 µL of 50 mM tris (2-carboxyethyl) phosphine (TCEP) and heating at 60°C for 1 h. Free thiol groups of cysteine residues were alkylated by adding 1 µL of 400 mM iodoacetamide and incubating for 30 min at room temperature in the dark with gentle agitation. Proteins were then digested overnight at 37°C with trypsin in 0.1 M TEAB pH 8.0 (protein/trypsin ratio 50:1 w/w).

### iTRAQ labeling and peptide fractionation by OFFGEL electrophoresis

The resulting peptides were tagged with the iTRAQ reagents Multiplex Kit (AB Sciex, Foster City, CA, USA). Each sample was labeled with one of two isobaric tags (114 for pFF+ and 115 for pFF-) reconstituted with 50 µL of isopropanol. The reaction was left to stand at room temperature for 60 min and then blocked by incubating with 8µL of hydroxylamine 5% for 15 min. The mixtures of labeled peptides were then pooled and dried under vacuum. The lyophilized peptides were dissolved in 800 µL of 5% CH_3_CN/ 0.1% formic acid (FA), and loaded (2 x 400µL) onto C18 Macro SpinColumns (Harvard Apparatus). Elution was performed with 2 × 200 µL of 50% CH_3_CN/ 0.1% FA. The samples were then dried under vacuum and dissolved in 360 µL of deionized water. A solution containing 6% glycerol and 0.3% IPG buffer pH 3-10 (Agilent, Santa Clara, CA, USA) was added to a final volume of 1.8 mL. Peptides were fractionated according to their pI on an Agilent 3100 OFFGEL fractionator using commercial 12 cm IPG pH 3-10 linear strips (GE Healthcare, Waukesha, WI, USA). The strips were rehydrated with 20 µL of rehydration solution (4.8% glycerol, 0.24% IPG buffer pH 3-10) per well. After a 30 min incubation, 150 µL of the sample solution were loaded per well. The isoelectric focalization was carried out at 20°C until a total voltage of 20 kV/h with a maximum current of 50 µA and a maximum power of 200 mW. After the focalization, peptide fractions (12/ for each group) were recovered in separate tubes and pH values were measured to check for the efficiency of the pH gradient. Fractions were then dried under vacuum, dissolved in 300 µL of 5% CH_3_CN/ 0.1% FA, and loaded (2 x 150 µL) onto C18 Micro SpinColumns (Harvard Apparatus). Elution was performed with 2 × 100 µL of 50% CH_3_CN/ 0.1% FA and eluted fractions were dried under vacuum and stored at -20 °C until MS analysis.

### Liquid chromatography-tandem mass spectrometry

Lyophilized peptides obtained from OFFGEL fractionation were dissolved in 8 µL of 5% CH_3_CN/ 0.1% FA; 5 µL of the resulting sample were injected for LC-MS/MS analysis. MS analysis was performed on a LTQ Orbitrap Velos Pro from Thermo Electron (San Jose, CA) equipped with a NanoAcquity UPLC system from Waters (Milford, MA, USA). Peptides were trapped on a home-made (5 µm 200 Å Magic C18 AQ 0.1 × 2 mm) pre-column (Michrom, Auburn, CA, USA) and separated on a home-made (5 µm 100 Å Magic C18 AQ, 0.75 × 15 mm) column (Michrom). The analytical separation was run for 65 min using a gradient of 99.9% H_2_O/ 0.1% FA (solvent A) and 99.9% CH_3_CN/ 0.1% FA (solvent B). The gradient was run as follows: 0–1 min 95% A and 5% B, then to 65% A and 35% B at 55 min, and 20% A and 80% B at 65 min at a flow rate of 220 nL/min. For MS survey scans, the OT resolution was set to 60000 and the ion population was set to 5 × 105 with an m/z window from 400 to 2000. A maximum of 3 precursors was selected for both the collision-induced dissociation (CID) in LTQ and the high-energy C-trap dissociation (HCD) with analysis in the OT. For MS/MS in the LTQ, the ion population was set to 7 x 103 (isolation width of 2 m/z) while for MS/MS detection in the OT, it was set to 2 × 105 (isolation width of 2.5 m/z), with resolution of 7500, first mass at m/z = 100, and maximum injection time of 750 ms. The normalized collision energies were set to 35% for CID and 60% for HCD. 

### Data extraction, database interrogation and relative protein quantification

Peak lists were generated from raw data using the embedded software from the instrument vendor (extract_MSN.exe v5.0). After peaklist generation, the CID and HCD spectra were merged for simultaneous identification and quantification by using EasyProtConv [17]. The merged mgf files, combined from the 12 analyzed OFFGEL fractions, were used for protein identification and quantification with EasyProt software platform v2.2 [17]. For protein identification, parameters were specified as follows: database = uniprot_sprot (2012_06 of 13-Jun-2012); taxonomy = *Homo Sapiens*; precursor error tolerance = 25 ppm; variable modification = oxidized methionine; ﬁxed modiﬁcations = carbamidomethylated cysteine, iTRAQ-labeled amino terminus and lysine; enzyme = trypsin; potential missed cleavage = 1; cleavage mode = normal; search round = 1, scoring model = CID_LTQ_scan_LTQ; instrument type = ESI-LTQ-Orbitrap. Protein and peptide scores were set up to maintain the false positive peptide ratio below 5%. For protein quantification, the isotopic correction was applied to reporter intensities according to the iTRAQ reagents certificate of analysis. iTRAQ reporter peak intensities were further normalized using the spiked LACβ standard. For each protein, the mean, the standard deviation, and the coefficient of variation (CV) of relative peptide intensities were obtained for the two experimental groups by using the EasyProt Mascat quantification module that computes a per-peptide ratio from the reporter ion abundance values for the given peptide [17]. The ratio of a protein is then computed as the geometric mean of all peptide ratios belonging to the protein. A Student's t-test distribution, with a null hypothesis stating that the log_2_ of the protein ratio is equal to zero (confidence interval=95%) was computed by the algorithm. Proteins featuring a ratio outside the interval of confidence, excluding zero, were considered as differentially expressed.

### Bioinformatics analyses

Proteins identified in the pFF+ and pFF- samples were imported into the Ingenuity Pathways Analysis software (IPA, Ingenuity Systems Inc., Redwood City, CA, USA) for batch analysis and identification of the canonical pathways as previously reported [18-20]. An assessment of significantly enriched processes networks for differentially expressed proteins was performed by evaluating the probability of a random intersection between the differentially expressed proteins with functional processes by applying the hypergeometric test [19]. GO term enrichment analysis was performed by using the DAVID software v6.7 (Database for Annotation, Visualization and Integrated Discovery) [21,22].

### Enzyme immunoassay for the assessment of complement functional activity

The activation of the classical, mannose-binding lectin and alternative complement pathways was measured using commercially available Wieslab complement system screen ELISA kit (Euro-Diagnostica, Malmo, Sweden), wherein individual pathways are activated by specific activators. Briefly, suggested dilutions of pFF+ and pFF- samples were added to microtiter wells precoated with IgM, mannan or LPS for classical, lectin and alternative pathways, respectively. The plate was incubated at 37°C for 1 h, and washed three times before addition of the conjugate (alkaline phosphatase-labeled anti–C5b-C9). The plate was further incubated at 22°C for 30 min and washed, and the color was developed by adding the substrate. The absorbance was read at 405 nm, and data were normalized as percent of inhibition according to manufacturer’s instruction.

### Fluorescent bead-based measurement of cytokines and growth factor in hFFs

Single hFF samples were simultaneously screened for the concentration of interleukin (IL)-1α, IL-1β, IL-2, IL-3, IL-4, IL-5, IL-6, IL-8, IL-9, IL-10, IL-12, IL-13, IL-15, IL-17, Eotaxin, Granulocyte-colony stimulating factor (G-CSF), Granulocyte-macrophage colony stimulating factor (GM-CSF), Interferon (IFN)-γ, IFN-γ inducible protein 10 (IP-10), Monocyte chemoattractant protein 1 (MCP-1), Macrophage inflammatory protein 1-alpha/beta (MIP-1α, MIP-1β), RANTES, Tumor necrosis factor alpha (TNF-α), Platelet-derived growth factor (PDGF-bb), Vascular endothelial growth factor (VEGF) and basic Fibroblast growth factor (bFGF). Data were acquired using a Bio-Plex 200 system equipped with Bio-Plex Manager software v5.0 (BioRad). All washing steps were performed on the Bio-Plex magnetic wash station (BioRad). Measurements were performed on individual hFF samples diluted (1:2) using the Bio-Plex 27-plex human cytokine kit from BioRad according to the manufacturer’s protocol. The standard curves optimization and the calculation of analyte concentrations were performed by using the Bio-Plex Manager software. A two-tailed t-test was used to test the significance between the FF+ and FF- groups by using the GraphPad Prism software v5.00 (La Jolla, CA USA).

## Results

### Quantitative proteomic analysis by iTRAQ labeling

In order to perform a comprehensive quantitative proteomic comparison of hFFs grouped on the basis of IVF outcome, an iTRAQ labeling-based strategy was exploited. To reduce the dynamic range of the samples and increase the probability of identifying low-abundance proteins, hFFs from women undergoing IVF were first depleted of human serum albumin and the major subclasses of gamma globulin (IgG). Depleted protein samples were monitored by analytical SDS-PAGE (data not shown) and pooled on the basis of IVF outcome. pFF+ and pFF- pooled samples were then digested with trypsin and labeled with the iTRAQ reagents. The resulting labeled peptide mixtures were then combined and fractionated according to their pI by using OFFGEL electrophoresis, as described in the Methods section. The fractionation quality was monitored by measuring the experimental pH values of individual OFFGEL fractions. A minimum overlap was further detected in the distribution of identified peptides along the OFFGEL fractions, thus confirming the efficiency of the method (Figure S1). Each of the 12 fractions/ per group was analyzed by high-resolution LC-MS/MS. Overall, 1529 unique peptides were assigned to 300 proteins by the EasyProt algorithm (Table S1). Isobaric quantification was further performed allowing the quantification of 89 non-redundant proteins with a minimum of two peptides per protein and a confidence threshold of 95% (p<0.05). Among them, 30 proteins showed a significant increased (≥1.5) or decreased (≤0.6) ratio and were considered as differentially expressed in pFF+ versus pFF- samples (Table 1). In particular, only 2 proteins were found to be down-regulated (i.e. actin cytoplasmic 1 and tubulin polyglutamylase) while 28 proteins were up-regulated in hFFs with positive IVF outcome. 

**Table 1:** List of the proteins differentially expressed between pFF+ and pFF- samples by LC-MS/MS analysis^a^

**Accession**	**IPA Symbol**	**Protein name**	**# Pep**	**pFF+/pFF-**	**p-value**	**Biological process**
P60709	ACTB	Actin, cytoplasmic 1	2	0.4	0.000887	Structural constituent of cytoskeleton
P01008	SERPINC1	Antithrombin-III (ATIII)	94	1.5	1.27E-57	Blood coagulation
Q9UBR2	CTSZ	Cathepsin Z	7	1.5	2.82E-06	Proteolysis
P00748	F12	Coagulation factor XII	10	2.0	5.05E-09	Blood coagulation
P0C0L5	C4B	Complement C4-B	110	1.6	9.69E-74	Complement activation
P13671	C6	Complement component C6	5	1.6	9.18E-06	Complement pathway
P02748	C9	Complement component C9	7	1.5	4.19E-07	Complement alternate pathway
P00751	CFB	Complement factor B	24	1.79	2.85E-27	Complement alternate pathway
P02671	FGA	Fibrinogen alpha chain	35	1.9	9.39E-42	Blood coagulation
P02675	FGB	Fibrinogen beta chain	23	1.7	4.09E-18	Blood coagulation
P02679	FGG	Fibrinogen gamma chain	11	2.0	1.22E-15	Blood coagulation
P05546	SERPIND1	Heparin cofactor 2	16	1.9	3.77E-20	Blood coagulation
P01861	IGHG4	Ig gamma-4 chain C region	34	2.5	2.93E-05	Innate immune response
P35858	IGFALS	Insulin-like growth factor-binding protein complex ALS	10	1.8	7.18E-15	Cell adhesion
Q14624	ITIH4	Inter-alpha-trypsin inhibitor heavy chain H4	43	1.5	6.80E-26	Inflammatory response
P14923	JUP	Junction plakoglobin	2	2.4	0.001659	Cell adhesion
P13645	KRT10	Keratin, type I cytoskeletal 10	46	3.7	1.5E-52	Structural constituent of cytoskeleton
P35527	KRT9	Keratin, type I cytoskeletal 9	40	3.6	4.76E-44	Structural constituent of cytoskeleton
P04264	KRT1	Keratin, type II cytoskeletal 1	66	4.0	9.14E-80	Structural constituent of cytoskeleton
P35908	KRT2	Keratin, type II cytoskeletal 2 epidermal	35	4.0	2.94E-34	Keratinization
P01042	KNG1	Kininogen-1	39	1.5	9.03E-27	Blood coagulation
Q96PD5	PGLYRP2	N-acetylmuramoyl-L-alanine amidase (PGRP-L)	6	1.5	0.001605	Innate immune response
Q7Z628	NET1	Neuroepithelial cell-transforming gene 1 protein	3	1.6	9.52E-06	Regulation of cell growth
P15309	ACPP	Prostatic acid phosphatase	11	1.8	3.57E-15	Nucleotide metabolic process
P00734	F2	Prothrombin	37	1.8	9.35E-45	Acute-phase response
P02753	RBP4	Retinol-binding protein 4	9	1.7	8.08E-12	Transport
P05452	CLEC3B	Tetranectin	9	1.7	1.27E-16	Plasminogen activation
Q6ZT98	TTLL7	Tubulin polyglutamylase	5	0.4	7.48E-05	Cell differentiation
P04070	PROC	Vitamin K-dependent protein C	2	1.5	0.005845	Blood coagulation
Q7Z7L9	ZSCAN2	Zinc finger and SCAN domain-containing protein 2	2	1.7	6.7E-05	Transcription regulation

^a^ Relative protein quantification was performed using iTRAQ reagents and LC-MS/MS. Differential expression was defined by a relative abundance ratio ≥1.5 and ≤0.6. Biological processes were assigned on the basis of Gene Ontology and UniProt databases.

### Bioinformatic Analysis of Differentially Expressed hFF Proteins Associated with IFV Outcome

To investigate the functional annotation of proteins quantified in hFFs, a gene ontology enrichment analysis was performed by using the DAVID Functional Annotation tools. Statistically significant GO annotations of biological process, molecular function and cellular component are reported in Figure S2. The clusterization according to biological processes revealed that most of the identified proteins were involved in defense and immune responses, inflammatory response and response to wounding. As would be expected in a fluidic environment, the classification based on cellular localization revealed that the largest populations of identified proteins belong to the extracellular space. Subcategories of vesicular compartments were also present. Furthermore, among significant enriched molecular function annotations, proteins endowed with both peptidase and peptidase inhibitor activity as well as enzyme and lipid binding capability were detected.

**Figure 1:**
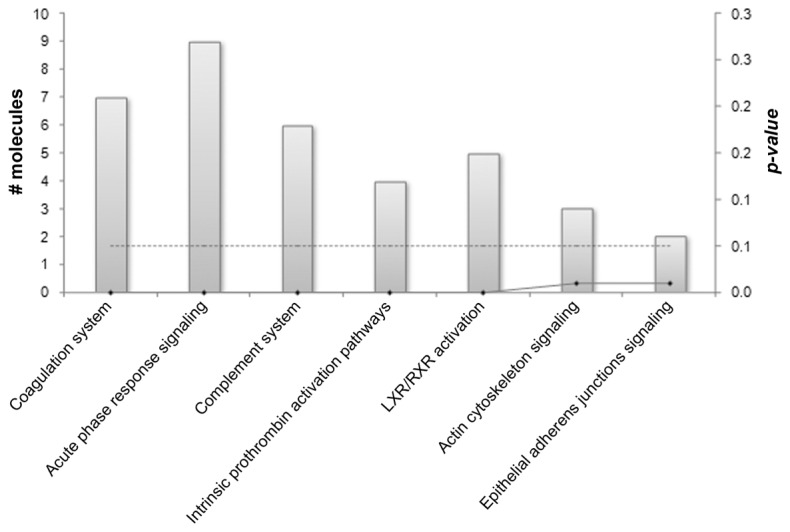
Bar chart of the enriched biological functions of differentially expressed proteins in pFF+ versus pFF- samples. The assessment of significantly enriched biological functions for differentially expressed proteins was performed by IPA software. p-values and the number (#) of molecules mapped on the enriched categories are reported.

**Figure 2:**
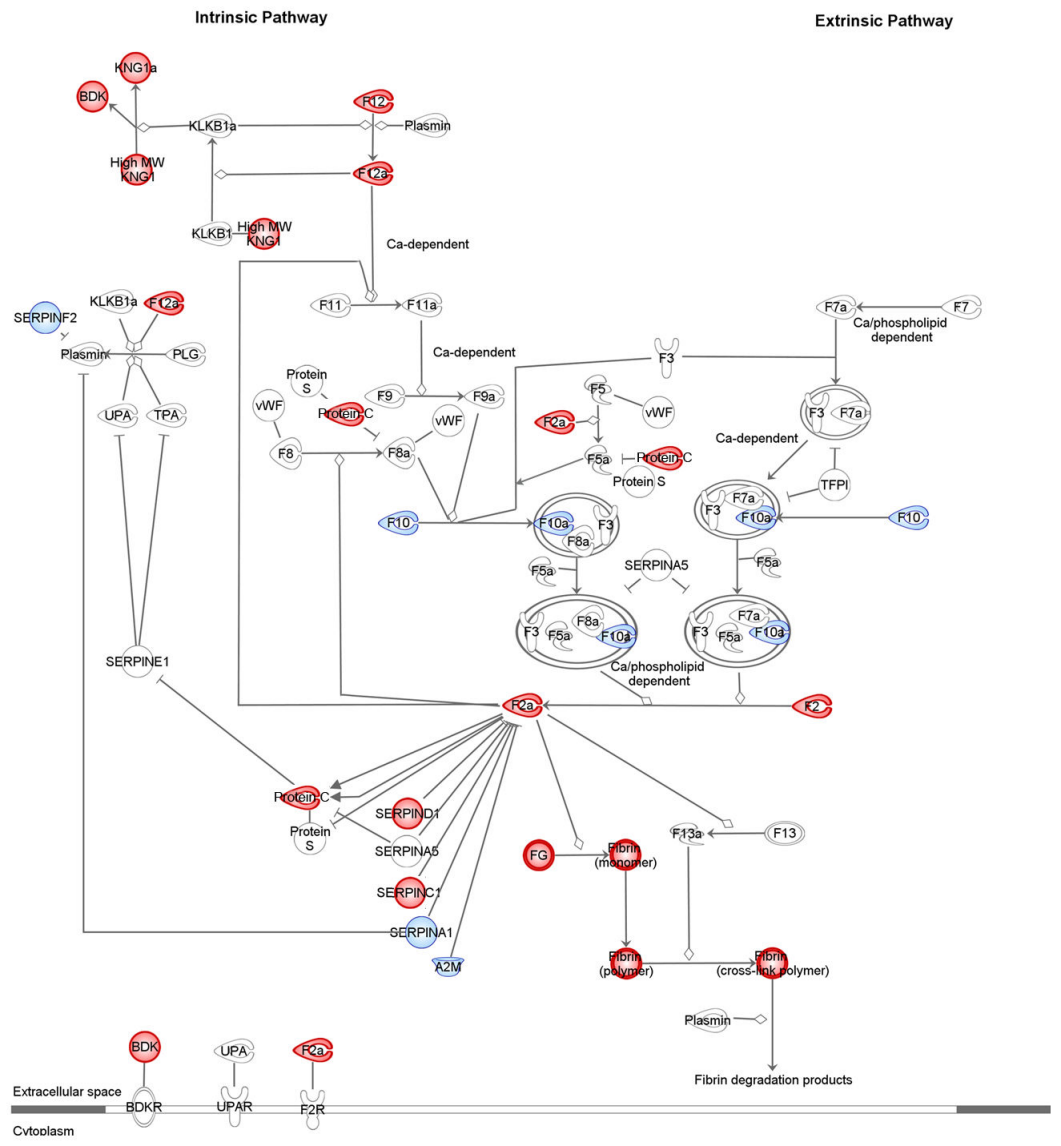
Mapping of a subset of differentially expressed proteins in pFF+ versus pFF- samples on the coagulation system pathway. According to IPA categorization, up-regulated proteins are coloured in red. The assessment of significantly enriched processes networks for differentially expressed proteins was performed by evaluating the probability of a random intersection between the differentially expressed proteins with functional processes by applying the hypergeometric test. The pathway components identified by the algorithm or with no significant differences in their expression levels are reported in white and blue, respectively. Molecules are named according to IPA software.

**Figure 3:**
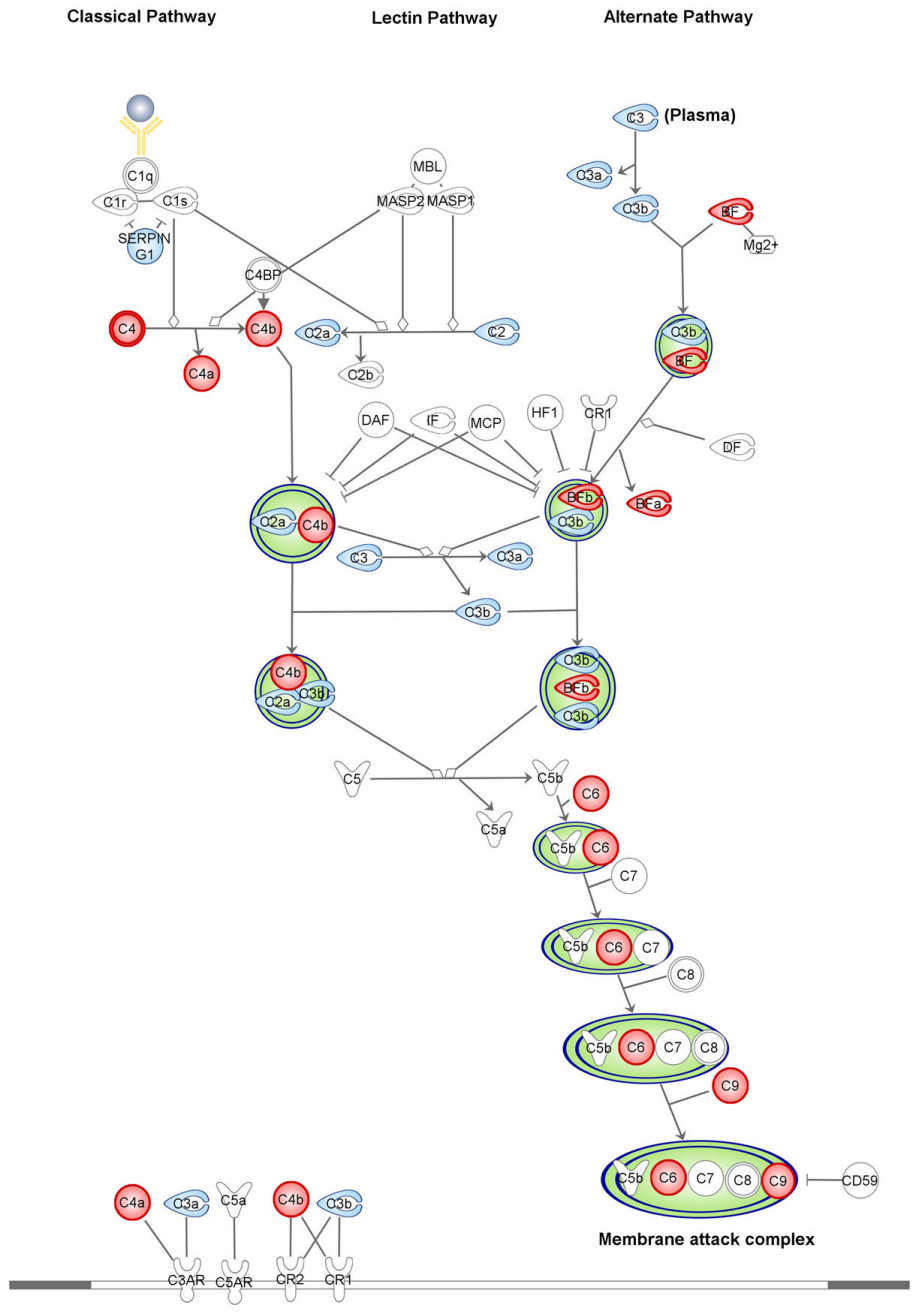
Mapping of a subset of differentially expressed proteins in pFF+ versus pFF- samples on the complement system canonical pathway. According to IPA categorization, up-regulated proteins are coloured in red. The assessment of significantly enriched processes networks for differentially expressed proteins was performed by evaluating the probability of a random intersection between the differentially expressed proteins with functional processes by applying the hypergeometric test. The pathway components identified by the algorithm or with no significant differences in their expression levels are reported in white and blue, respectively. Molecules are named according to IPA software.

**Figure 4:**
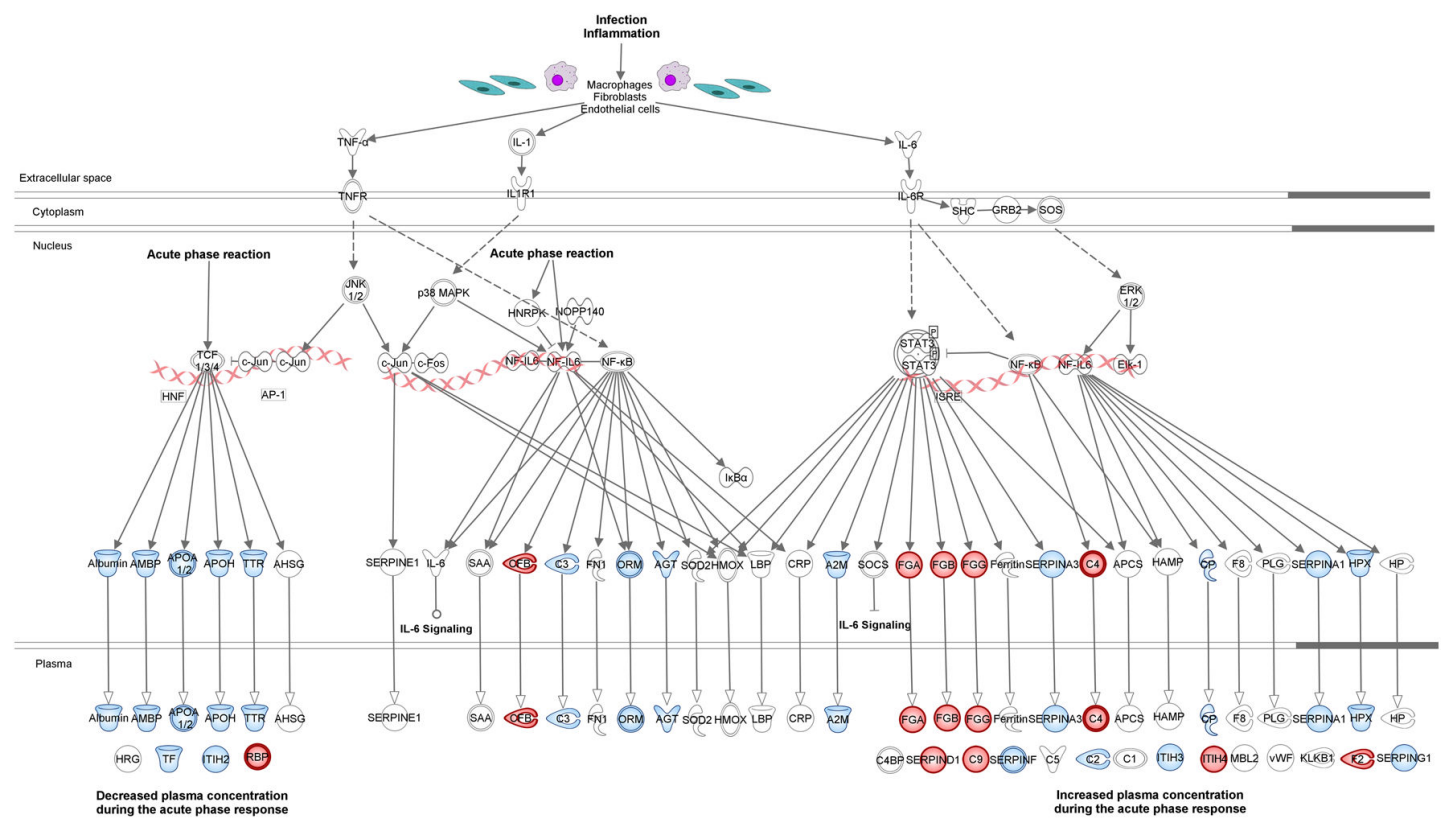
Mapping of a subset of differentially expressed proteins in pFF+ versus pFF- samples on the acute phase response signaling canonical pathway. According to IPA categorization, up-regulated proteins are coloured in red. The assessment of significantly enriched processes networks for differentially expressed proteins was performed by evaluating the probability of a random intersection between the differentially expressed proteins with functional processes by applying the hypergeometric test. The pathway components identified by the algorithm or with no significant differences in their expression levels are reported in white and blue, respectively. Molecules are named according to IPA software.

The subsequent pathway analysis revealed that a significant number of differentially regulated proteins were involved in biological functions related to blood coagulation, acute phase response signaling, prothrombin activation and complement system (Figure 1). The majority of these proteins were mapped on four main IPA canonical pathways. In particular, several proteins were mapped on the coagulation system (Figure 2) and the prothrombin activation (Figure S3) pathways. In addition, proteins of the complement system (Figure 3) and acute phase response signaling (Figure 4) pathways were found up-regulated in pFF+ versus pFF- samples. 

### Validation of complement functional activation by Enzyme immunoassay

On the basis of the iTRAQ results and the relevance of complement cascade as a fundamental immune surveillance system, the functional activity of the complement activation was assessed. To this aim, the determination of the classical, alternative and lectin pathways was performed by using an ELISA-based procedure widely used to test the complement activation in human serum. As a result, a higher activation of the classical complement pathway was revealed in the pFF+ (40.5%) versus pFF- (14.6%) samples (Figure S4) thus confirming iTRAQ results related to the up-regulation of proteins belonging to the complement system. No activation of both complement alternative and lectin pathways was detected in the hFF samples under investigation.

### Analysis of hFF cytokines and growth factors profiles by multiplexed flow cytometric protein array

The expression levels of 27 cytokines in individual hFF were determined by using a multiplexed assay. A categorization of analytes into pro-inflammatory and anti-inflammatory cytokines, chemokines and growth factors was made on the basis of their *in vivo* and *in vitro* activities. No significant differences were detected in the expression levels of growth factors (i.e. bFGF, IL-7, G-CSF, GM-CSF, PDGF-bb, VEGF, Figure S5) and anti-inflammatory cytokines (i.e. IL-1ra, IL-4, IL-10, IL-13, Figure S6). 

**Figure 5:**
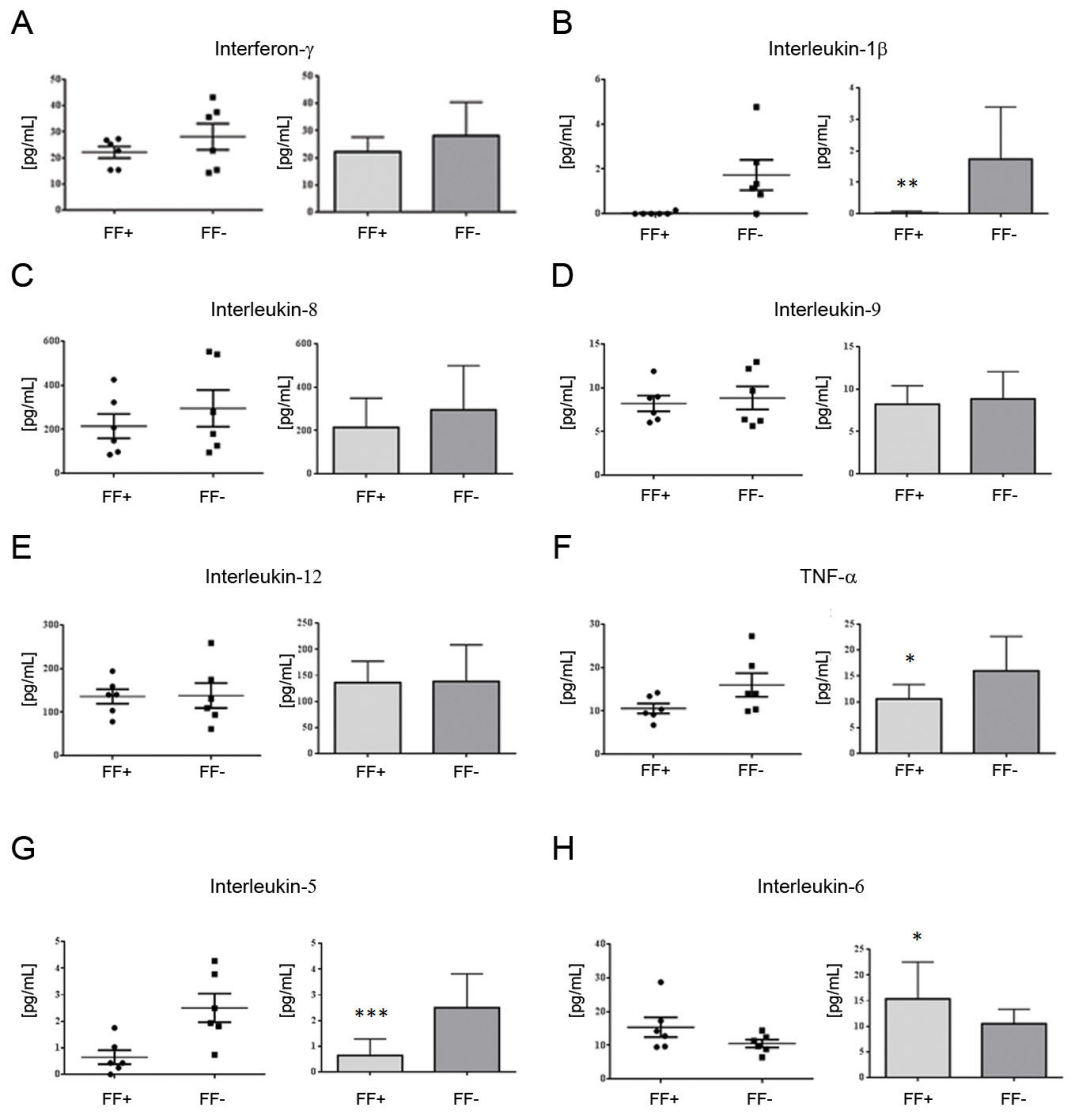
Expression levels of pro-inflammatory cytokines in hFF samples. hFF samples were simultaneously screened for the concentration of the pro-inflammatory cytokines IFN-γ (A), IL-1β (B), IL-8 (C), IL-9 (D), IL-12 (E), TNF-α (F), IL-5 (G) and IL-6 (H) by using the Bio-Plex 27-plex human cytokine kit from BioRad according to the manufacturer’s protocol. Measurements were performed on individual hFF samples diluted (1:2) using the standard curves optimization and the calculation of analyte concentrations of the Bio-Plex Manager software. Data are reported as scatter plots and average concentrations. * p-value ≤ 0.1; ** p-value≤ 0.05; *** p-value ≤ 0.01 FF+ *versus* FF- dataset.

**Figure 6:**
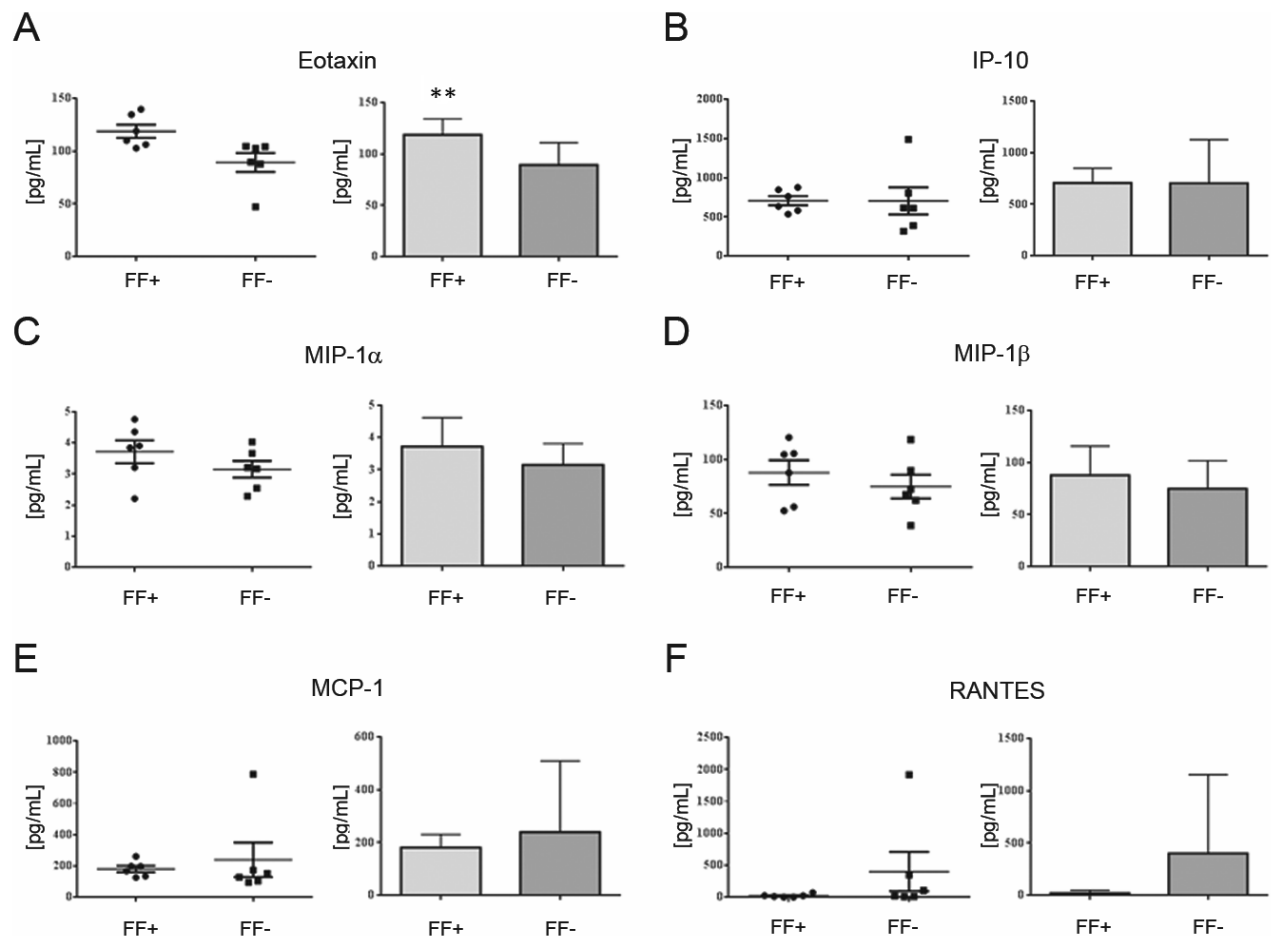
Expression levels of chemokines in hFF samples. hFF samples were simultaneously screened for the concentration of the chemokines Eotaxin (A), IP-10 (B), MIP-1α (C), MIP-1β (D), MCP-1 (E) and RANTES (F) by using the Bio-Plex 27-plex human cytokine kit from BioRad according to the manufacturer’s protocol. Measurements were performed on individual hFF samples diluted (1:2) using the standard curves optimization and the calculation of analyte concentrations of the Bio-Plex Manager software. Data are reported as scatter plots and average concentrations. ** p-value≤ 0.05 FF+ *versus* FF- dataset.

Among pro-inflammatory cytokines (i.e. INF-γ, IL-1β, IL-5, IL-8, IL-9, IL-12 and TNF-α, Figure 5), a lower level of IL-1β (p-value ≤ 0.05), IL-5 (p-value ≤ 0.01) and TNF-α (p-value ≤ 0.1) was found in FF+ when compared to the FF- dataset 2 cytokines (i.e. IL-2 and IL-6) included in the Bio-Plex protein array panel, are considered to have a dual role, being endowed with both pro- and anti-inflammatory activities under different conditions. IL-2 as well as IL-17 and IL-15 were not detected in both FF+ and FF- groups. The levels of IL-6 were higher (p-value ≤ 0.1) in FF+ when compared to the FF- dataset (Figure 5H). No significant differences were finally revealed for chemokines (i.e. IP-10, MIP-1α, MIP-1β, MCP-1 and RANTES, Figure 6) with the only exception of Eotaxin, for which a higher concentration in FF+ samples (p-value ≤ 0.05) was detected. 

## Discussion

Multiplexed high-throughput *-omics* strategies provide an integrated and broader view of biological regulatory networks and pathways [23]. This aspect is of pivotal importance since protein activity is dependent not only on its abundance, but is also influenced by the effects of interacting, modifying, antagonistic and synergistic proteins [23]. In-depth investigations of protein networks, pathways and dynamics are eliciting a growing interest for their potential implications in biomarker discovery and clinical practice [23]. However, the high dynamic range of biological systems makes the study of complex matrices, such as biological fluids, especially challenging. In the last years, multiplexed immunoassays for the simultaneous measurement of low-abundance proteins (e.g. cytokines, chemokines, growth factors) in complex biological matrices have become significant tools for quantitative studies in biomarker and diagnostic discovery, thus supporting and integrating proteomic surveys by MS approaches [24-28]. 

In this work, an iTRAQ labeling strategy has been complemented with a multiplexed protein array approach to analyze hFFs with the aim to investigate biological processes and pathways associated to IVF outcome. This combined strategy allowed the identification of candidate proteins that potentially correlate with IVF outcome, several of which are components of the coagulation and complement systems. 

Consistent with our findings are previous studies reporting that complement cascade components in hFF may play a key role in reproductive processes [14-16]. A comparative study aimed at identifying differences in protein composition of hFFs from women undergoing successful IVF with respect to plasma, revealed that a majority of differentially expressed proteins belonged to the complement cascade (i.e. complement C4-A, complement C3, complement component C9). In addition, several regulatory proteins of the complement system have also been described as differentially expressed (clusterin, complement factor H, ficolin-3) [15]. It has been hypothesized that inhibition of the complement system is required for maintaining oocyte viability and that the lack of this inhibition may lead to miscarriage [16]. Although the specific role of the complement system in pregnancy is still a matter of debate, it has been reported that normal human pregnancy is associated with physiologic complement activation acting as a compensatory mechanism aimed to protect the host against infection [29]. Accordingly, in the present study, several components of the complement system (e.g. complement component C6, complement component C9, complement component C4B) were up-regulated in the pFF+ versus pFF- samples. The increased expression level of several members of the complement cascade prompted us to perform a validation of complement functional activation. Our results attest a higher activation of the classical complement pathway in pFF+, while no activation of complement alternative and lectin pathways was detected. Interestingly, the complement system, traditionally depicted as a linear cascade of separate pathways, is now considered a hub-like network that acts as a global mediator in immune surveillance, cell and tissue homeostasis and repair which is tightly connected to other systems [30,31]. Although it has long been known that proteases such as plasmin, thrombin, elastase and plasma kallikrein can cleave and activate C3, this extrinsic protease pathway has gained more attention in the context of reciprocal crosstalk between complement and coagulation. Complement, indeed, amplifies coagulation and inhibits fibrinolysis. Similarly, some components of the coagulation cascade amplify complement activation [30]. 

Several members of the coagulation systems were found up-regulated in the pFF+ versus pFF-, including fibrinogen, kininogen-1, prothrombin and coagulation factor XII. It has been reported that activated factor XII can activate the classical complement pathway through C1 cleavage [32], whereas thrombin directly cleaves C5 and generates biologically active C5a [33]. Follicular fluid contains members of the coagulation system and, upon ovulation, undergoes clotting within the ruptured follicle [34]. Based on the complexity of coagulation, it has been proposed that multiple mechanisms are involved in the regulation of follicular fluid clotting depending on the female reproductive cycle [34]. Indeed, an anticoagulant state initially exists during follicle growth and rupture (i.e. follicular and ovulatory phases, respectively). Prevention of clotting following ovulation ensures that a liquid state is initially maintained for the oocyte to be successfully delivered to the oviduct. During the luteal phase, a procoagulant state is established, leading to the formation of fibrin clots that support the migration of luteal cells and the formation of the *corpus luteum*. There are also evidences that human follicular fluid is markedly hypocoagulable with respect to plasma, due to suppression of the anticoagulant effect of thrombin by endogenous inhibitory mechanisms [34,35]. In line with these findings, compared with plasma, hFF contains higher levels of tissue factor pathway inhibitor and lower concentrations of factors V and VIII, which might limit the amplification of thrombin formation [35-37]. In this study, several members affecting the pro- and/or anti-coagulant state of hFF were identified, including three thrombin inhibitors up-regulated in pFF+ versus pFF- samples (i.e. antithrombin-III, vitamin K-dependent protein C and heparin cofactor 2). 

Although the biological effect of the coagulation cascade components within hFF and their interaction with other pathways remain to be unraveled, numerous evidences points to intricate crosstalk between coagulation and inflammation, whereby both systems are able to considerably affect and modulate each other [38]. Indeed, coagulation factors, fibrin and fibrinolytic proteins and anticoagulant pathways are able to affect cytokines production and inflammatory cell activity. Several previous reports suggest that ovulation can be compared to an inflammatory event, also based on the presence of a large amount of acute-phase response proteins in hFF [8,15]. This hypothesis is also supported by the presence in hFF of inflammatory mediators, such as pro-inflammatory interleukins either deriving by ovarian local synthesis and/or released during follicular maturation [4]. Our results showed that several differentially expressed proteins, up-regulated in pFF+ versus pFF- samples, were mapped on the acute phase response signaling pathway. Based on this finding, we decided to investigate the expression profiles of 27 soluble factors including cytokines, chemokines and growth factors, most of which are key modulators of inflammation processes. In the ovary, cytokines are involved in several physiological processes including tissue remodeling during ovulation, follicular growth, steroidogenesis and recruitment and activation of leukocytes necessary for ovulation [39]. Several studies explored the potential of determining cytockines and growth factors as biochemical predictors of oocyte quality, infertility etiology, IVF success and embryos implantation potential [4,40-43]. Although contradictory results have been reported concerning a possible correlation of some cytokines and chemokines with female fertility, the importance of understanding their role in human reproduction and their relevance to IVF outcome is undoubted. 

In our study, we did not observe massive changes in the cytokines profile of hFFs grouped on the basis of IVF outcome. However, it is interesting that three cytokines for which differentially expression was observed (i.e. IL-1β, IL-6 and TNF-α) were integrated as upstream regulators in the acute phase response signaling pathway previously pointed out by quantitative proteomic analysis (Figure 4). In particular, within this pathway, no substantial expression changes were revealed for the downstream effectors of IL-1β and TNF-α, both down-regulated in the FF+ dataset. In contrast, the higher levels of IL-6 detected in FF+ samples were consistent with the increased levels of target proteins acting as downstream effectors of the acute phase response pathway also found up-regulated by the iTRAQ approach (Figure 4). Several evidences have accumulated that IL-6 can directly influence ovarian function as an important regulator in the reproductive processes [39,44]. Furthermore, while the roles of TNF-α in female reproduction is still a matter of debate based on its complex and dynamic involvement in different stages of folliculogenesis [45,46], previous studies reported that lower levels were detected in hFF whose oocytes were able to generate better embryos and successful IVF outcome [47].

Overall, our work explores, for the first time on hFFs, the high potentiality of applying integrated and complementary strategies capable of accurately quantifying proteins with remarkable differences in their abundance in a complex matrix. This approach is particularly useful in order to coalesce knowledge from *-omics* data to better understand inter-relationships and crosstalk between dynamic biological processes, thus extending the number of possible candidate biomarkers for targeted validation in order to promote clinical and translational research.

## Supporting Information

Figure S1 **A) Experimental pH values of OFFGEL fractions; B) Distribution of unique peptide sequences along OFFGEL fractions.**
(PPT)

Figure S2 **Enrichment analysis performed by the DAVID software for the identification of statistically significant gene ontology annotations of biological process, molecular function and cellular component of proteins quantified in hFFs.**
(XLS)

Figure S3 **Mapping of a subset of differentially expressed proteins in pFF+ versus pFF- samples on the prothrombin activation pathway.** According to IPA categorization, up-regulated proteins are coloured in red. The pathway components identified by the algorithm or with no significant differences in their expression levels are reported in white and blue, respectively. Molecules are named according to IPA software.
(TIF)

Figure S4 **Validation of classical, alternative and lectin complement functional activation by Enzyme immunoassay.**
(TIF)

Figure S5 **Expression levels of growth factors in hFF samples.** The concentrations of the growth factors bFGF (A), IL-7 (B), G-CSF (C), GM-CSF (D), PDGF-bb (E) and VEGF (F) were measured in individual samples and reported as scatter plots and average concentrations.
(TIF)

Figure S6 **Expression levels of anti-inflammatory cytokines in hFF samples.** The concentrations of the anti-inflammatory cytokines IL-1ra (A), IL-4 (B), IL-10 (C), IL-13 (D) were measured in individual samples and reported as scatter plots and average concentrations.
(TIF)

Table S1 **List of proteins identified by high-resolution LC-MS/MS by the EasyProt algorithm.**
(XLS)
