# Supplementary figures and images for: An Integrated Approach Based on Multiplexed Protein Array and iTRAQ Labeling for In-Depth Identification of Pathways Associated to IVF Outcome
Source: PLoS One. 2013 Oct 16;8(10):e77303. doi: 10.1371/journal.pone.0077303 (PMC3797768; doi:10.1371/journal.pone.0077303)

## Slide 1
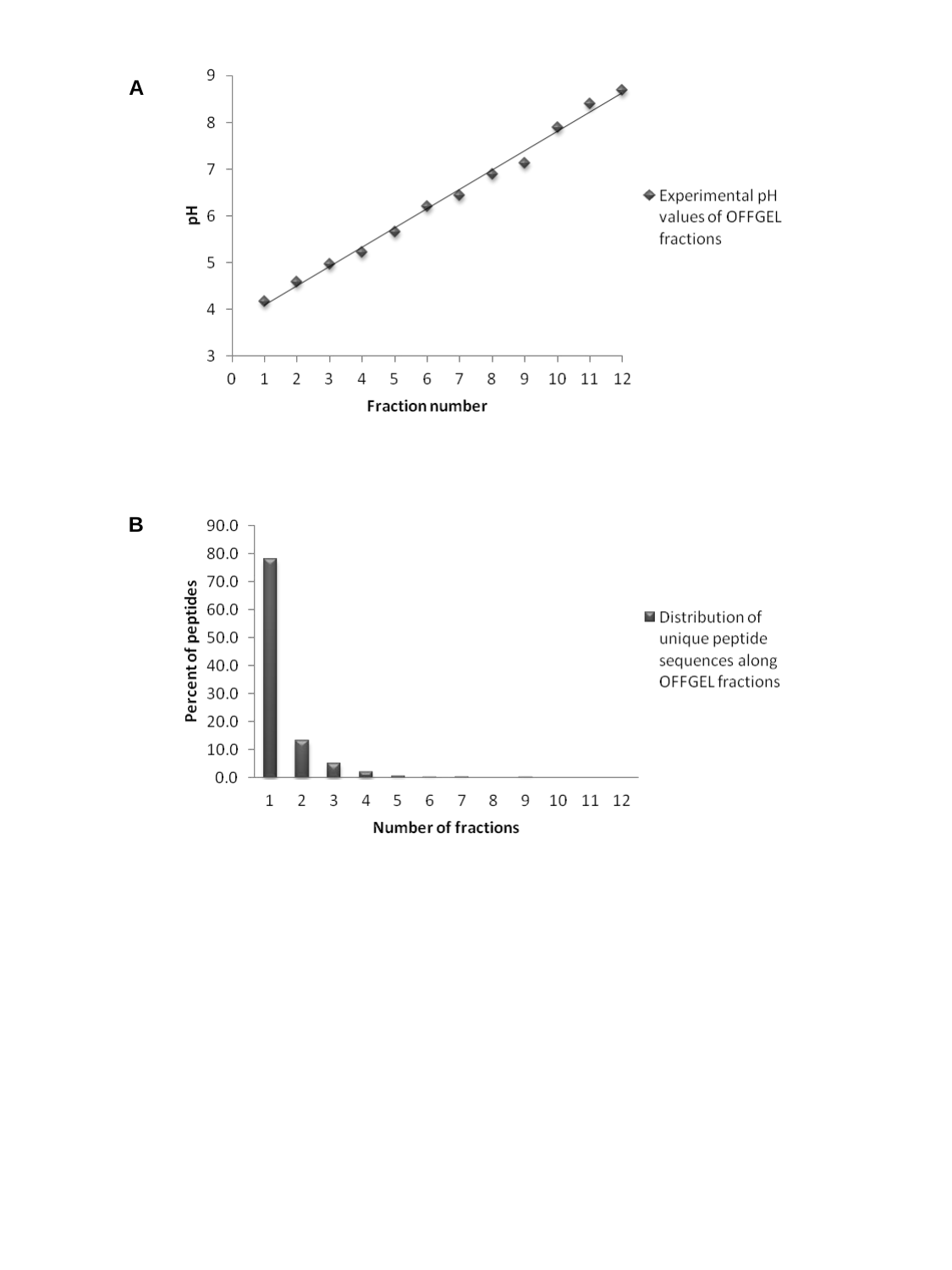

A
B

Supplement: Figure S1 — A) Experimental pH values of OFFGEL fractions; B) Distribution of unique peptide sequences along OFFGEL fractions. (PPT) [file pone.0077303.s001.ppt]

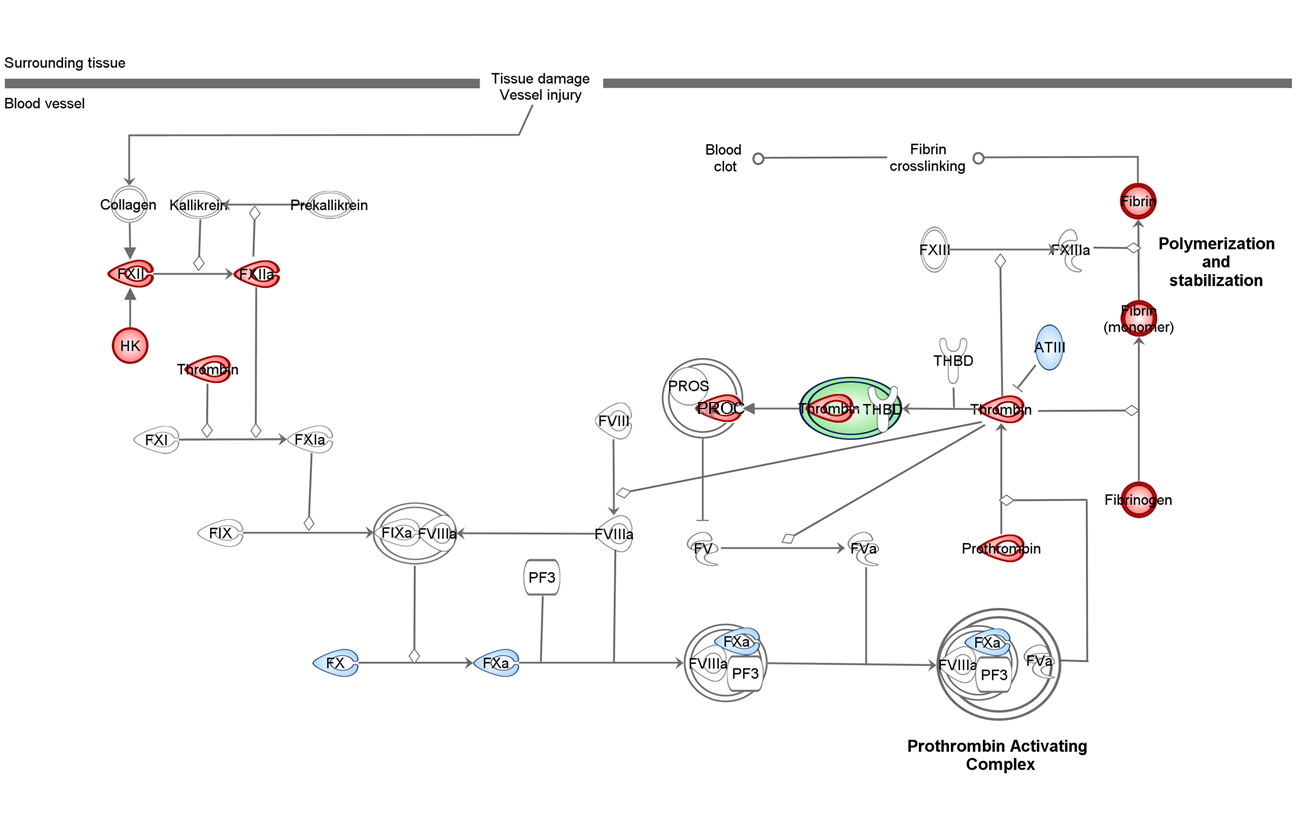

Supplement: Figure S3 — Mapping of a subset of differentially expressed proteins in pFF+ versus pFF- samples on the prothrombin activation pathway. According to IPA categorization, up-regulated proteins are coloured in red. The pathway components identified by the algorithm or with no significant differences in their expression levels are reported in white and blue, respectively. Molecules are named according to IPA software. (TIF) [file pone.0077303.s003.tif]

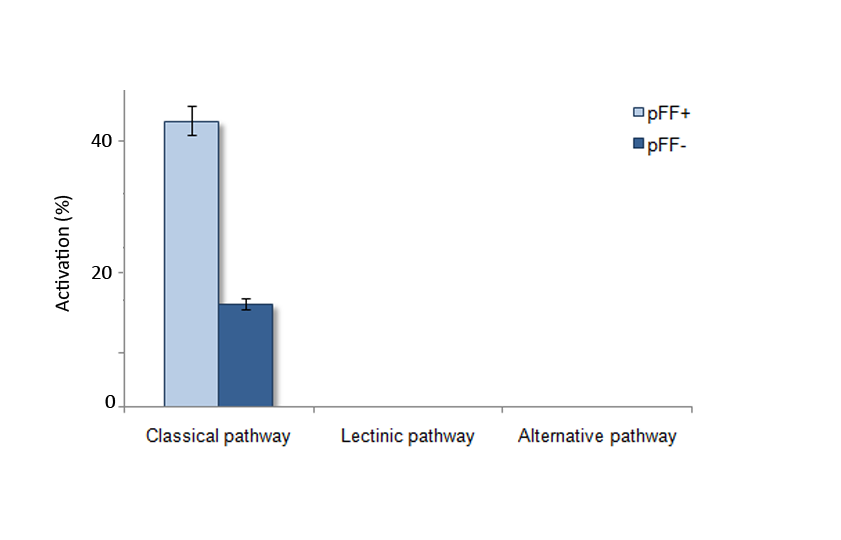

Supplement: Figure S4 — Validation of classical, alternative and lectin complement functional activation by Enzyme immunoassay. (TIF) [file pone.0077303.s004.tif]

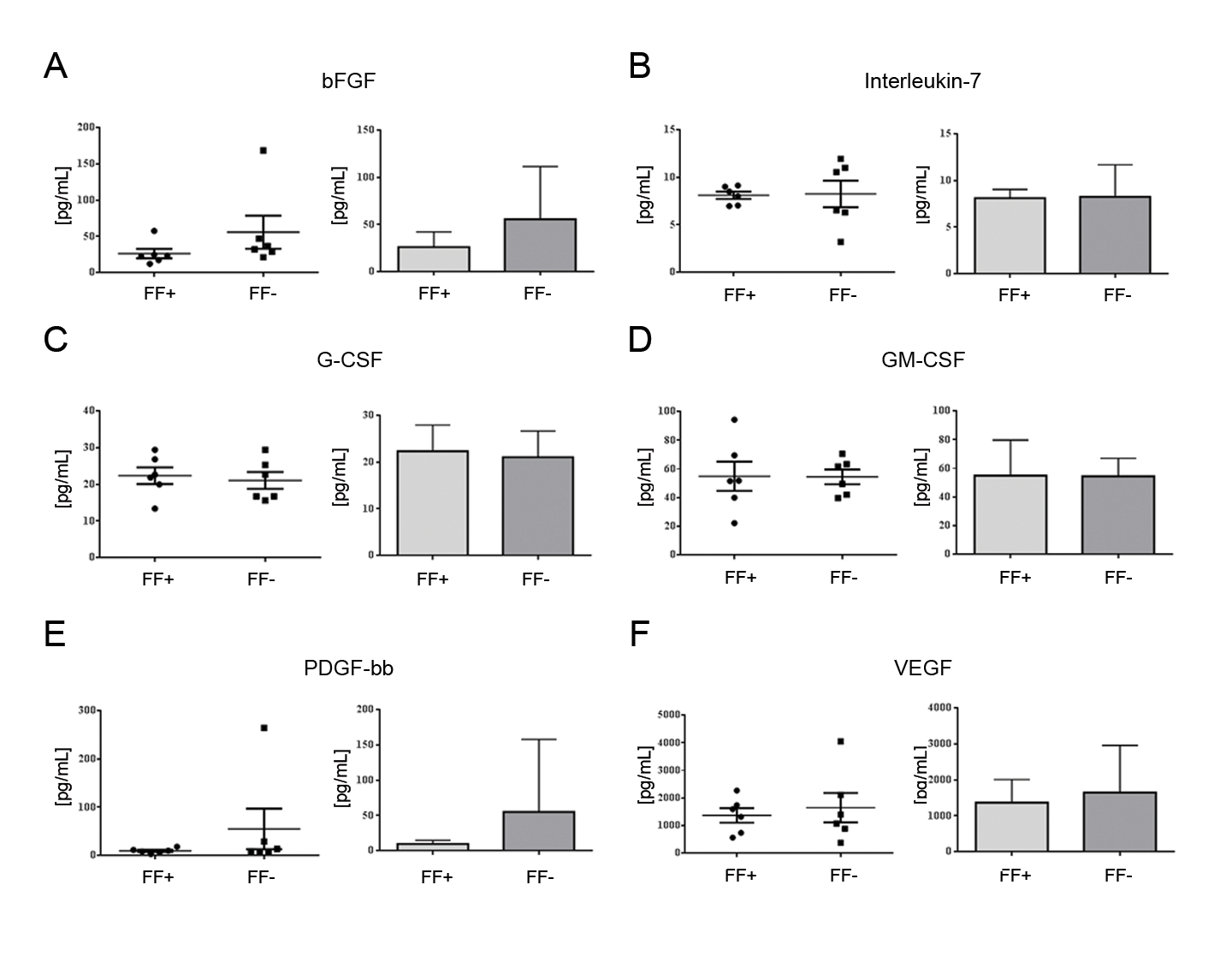

Supplement: Figure S5 — Expression levels of growth factors in hFF samples. The concentrations of the growth factors bFGF (A), IL-7 (B), G-CSF (C), GM-CSF (D), PDGF-bb (E) and VEGF (F) were measured in individual samples and reported as scatter plots and average concentrations. (TIF) [file pone.0077303.s005.tif]

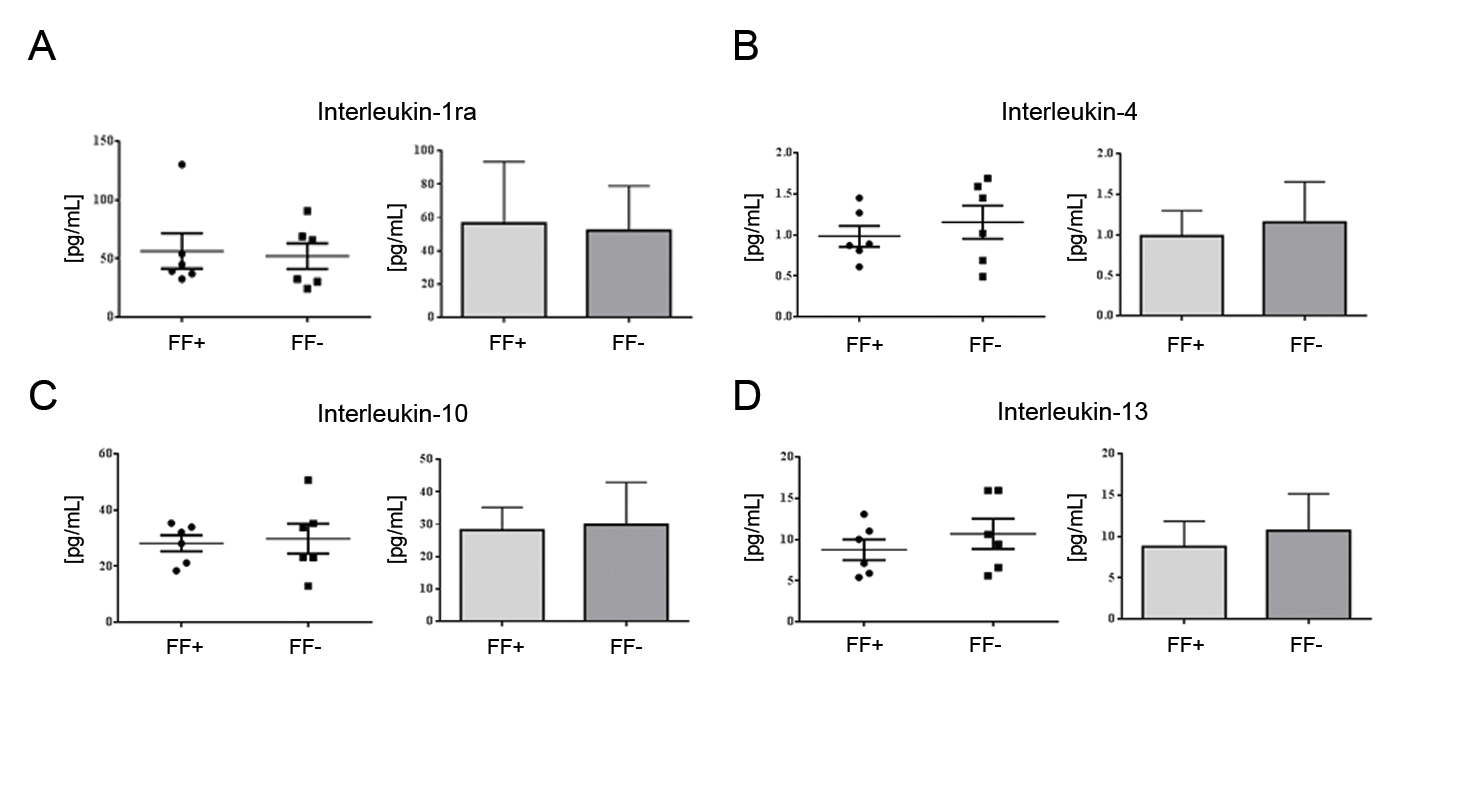

Supplement: Figure S6 — Expression levels of anti-inflammatory cytokines in hFF samples. The concentrations of the anti-inflammatory cytokines IL-1ra (A), IL-4 (B), IL-10 (C), IL-13 (D) were measured in individual samples and reported as scatter plots and average concentrations. (TIF) [file pone.0077303.s006.tif]
